# Personal budget: integration of social and health resources of frail elderly in supported discharge: Martina Giusti

**DOI:** 10.1093/eurpub/ckac131.007

**Published:** 2022-10-25

**Authors:** M Giusti, S Simoni, N Persiani

**Affiliations:** Experimental and Clinic Medicine, Università di Firenze, Florence, Italy

## Abstract

During COVID-19 pandemic, health and social condition of frail elderly got worse at worldwide level. This aggravation was due both to isolation and higher mortality rate for COVID-19, influenced by the widespread condition of comorbidity in this segment of population. The progressive change of needs in this target weren't matched with a prompt and adequate response, which could guarantee the taking charge of frail elderly. Personal budget is a tool for integrating public health, social assistance and family efforts on the international scene. In the Italian context, it is emerging as means to program and coordinate economic and professional, institutional, and personal resources, finalising them to provide a personalised pathway. Research aims to value the contribution of personal budget association to effectiveness and efficacy of personalised pathway for frail elderlies in early supported discharges. A research-intervention approach was done. Personal budget was applied to a significant sample of cases of personalised pathways for frail elderly in supported discharge in the Local Health District of Teramo. The study compared used resources and obtained health outcomes between the sample and a control group. The application of the personal budget permits the immediate maximisation of resources use and the quantification of the deviation between the available and the needed resources for provision of personalised pathway. Moreover, this tool supported the planning of services, the integration of health and social actions, the monitoring of adherence to planned programmes and the application of corrective mechanisms, if these are necessary, improving the health outcomes. Personal budget contributes to increase the integrated and continued taking charge of frail elderly in supported discharges in the short-medium term. Globally, this tool could be an organisational and economic answer for responding in a sustainable way to international increasing of life expectancy.

**Key messages:**

• Personal budget manages and controls both health and social activities and resources.

• Personal budget supports the establishment of an integrated and continuous taking charge system of the frail elderly.

